# Comment on “Kidney Function and Incident Stroke and Dementia Using an Updated Estimated Glomerular Filtration Rate Equation Without Race: The Multi-Ethnic Study of Atherosclerosis”

**DOI:** 10.1016/j.xkme.2025.101033

**Published:** 2025-05-19

**Authors:** Paul T. Williams

**Affiliations:** Molecular Biophysics and Integrated Bioimaging Division, Lawrence Berkeley National Laboratory, Berkeley, CA

To the Editor:

Data reported by Moen et al^1^ support the 2009 race-corrected glomerular filtration rate calculated using creatinine (GFRcr). Considering stroke and dementia as chronic kidney disease surrogates, their Table S5 data suggest that the incidence dementia rate in African Americans reclassified from eGFRcr ≥60 to <60 mL/min/1.73 m^2^ by the 2021 eGFRcr (11.4/1000 person-years) was more similar to those consistently >60 (5.6/1000 person-years) than those consistently <60 (23.3/1000 person-years), and that stroke incidence in those reclassified (3.24/1000 person-years) was more similar to those consistently ≥60 (3.73/1000 person-years) than those consistently <60 (7.53/1,000 person-years), suggesting the reclassification is unwarranted and the race-corrected formula is more appropriate (≥60 rates calculated as 60-90 and >90 incident rates weighted by person-years). Whereas Moen et al attribute the slightly higher hazard ratios for stroke and dementia outcomes for African Americans categorized using the 2009 race-corrected vis-à-vis the 2021 race-free eGFR to overestimating African American eGFR in the 2009 equation, it seems more likely that the higher hazard ratio for the race-adjusted formula reflects its greater accuracy, given that misclassification would attenuate, not intensify, the hazard ratios. For example, using kidney replacement therapy and death as chronic kidney disease surrogates, Yan et al^2^ showed both surrogates had higher rates for 2009 race-corrected (15.2 and 62.9 per 1,000 patient-years, respectively) than the 2021 race-free eGFRcr (10.5 and 51.6, respectively), the likely explanation being that reclassified African Americans diluted the associations.^3^^,^^4^ While praising the expanded treatment opportunities for 52 African Americans (7%) reclassified to eGFR <60, Moen et al are silent about the opportunities lost in 85 non-African Americans (34%) reclassified to eGFR ≥60 by the 2021 GFRcr.
